# Growth of Giant Peptide Vesicles Driven by Compartmentalized Transcription–Translation Activity

**DOI:** 10.1002/chem.202003366

**Published:** 2020-11-24

**Authors:** Thomas Frank, Kilian Vogele, Aurore Dupin, Friedrich C. Simmel, Tobias Pirzer

**Affiliations:** ^1^ Physics-Department and ZNN Technical University Munich Am Coulombwall 4a 85748 Garching Germany

**Keywords:** artificial cells, elastin-like polypeptides, membranes, synthetic biology, vesicle growth

## Abstract

Compartmentalization and spatial organization of biochemical reactions are essential for the establishment of complex metabolic pathways inside synthetic cells. Phospholipid and fatty acid membranes are the most natural candidates for this purpose, but also polymers have shown great potential as enclosures of artificial cell mimics. Herein, we report on the formation of giant vesicles in a size range of 1 μm–100 μm using amphiphilic elastin‐like polypeptides. The peptide vesicles can accommodate cell‐free gene expression reactions, which is demonstrated by the transcription of a fluorescent RNA aptamer and the production of a fluorescent protein. Importantly, gene expression inside the vesicles leads to a strong growth of their size—up to an order of magnitude in volume in several cases—which is driven by changes in osmotic pressure, resulting in fusion events and uptake of membrane peptides from the environment.

## Graphical abstract

**Encapsulation of RNA** transcription or cell‐free protein expression reactions inside vesicles made from elastin‐like polypeptide membranes promotes strong growth of the vesicles, which is driven by the generated osmotic pressure accompanied by multiple fusion events. Compared to other membranous compartments, the vesicles display extraordinary stability, which allows growth over several orders of magnitude in volume.

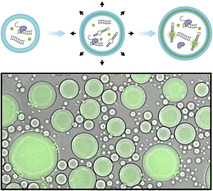

One of the most prominent goals of bottom‐up synthetic biology is the creation of synthetic cells.^[1]^ Such systems are envisioned to display a set of properties and capabilities that are associated with extant living cells, namely (i) compartmentalization, (ii) growth, self‐maintenance and self‐replication, (iii) signaling, communication and sensing, and (iv) the potential for evolution through replication and transfer of genetic information.

Compartmentalization is an essential prerequisite for the remaining properties, and has been achieved using a wide variety of different approaches.^[2]^ Cell‐scale reaction containers have been created from phospholipid membranes,^[3]^ using fatty acids^[4]^ or polymers,^[5]^ emulsion droplets,^[6]^ coacervates,^[7]^ or even using microfluidics‐based DNA chips.^[8]^

Several synthetic cell models already displayed at least some of the desired properties listed above. For instance, Szostak and co‐workers reported on fatty acid vesicles—serving as primordial cell models—, which were able to grow and divide upon external feeding with fatty acids.^[9]^ In further work, enzyme‐free copying of nucleic acid templates was shown inside fatty acid vesicles.^[10]^ Kurihara et al. succeeded to show DNA amplification inside lipid‐based giant vesicles, which were able to grow when membrane precursors were added to the outside solution.^[11]^ Growth was also observed for phospholipid vesicles externally fed with fatty acids, which contained a cell‐free protein synthesis reaction.^[12]^ Other research groups focused on in situ phospholipid biosynthesis inside of liposomes. For instance, Hardy et al. catalytically synthesized phospholipids from simpler precursors, which also resulted in membrane growth.^[13]^

More closely mimicking lipid synthesis in natural cells, Scott et al. established parts of the complex phospholipid synthesis pathway inside of liposomes. To this end, all the required enzymes were encoded on DNA templates and produced inside of the liposomes via cell‐free gene expression.^[3a]^ Due to their similarity to biological cell membranes, phospholipid membranes appear to be the most natural candidates for compartmentalization of synthetic cell‐mimicking systems. From a technical point of view, however, phospholipids have several drawbacks. For instance, phospholipids form membranes with a relatively high resistance to a change in membrane area, and even minor stretching causes membrane rupture.^[1b]^ Membranes composed of lipid mixtures can have more favorable mechanical properties, but *in vesiculo* production of mixed membranes would be even more challenging than for homogeneous membranes.

Other membrane‐forming molecules such as amphiphilic block co‐polymers or polypeptides represent an interesting alternative to phospholipids.[^1b^, ^14^] Such membranes are mechanically quite robust and even capable of storing elastic energy.^[15]^ Furthermore, membrane forming peptides can be easily produced inside of vesicles^[16]^ using cell‐free transcription‐translation systems.^[17]^ A particularly interesting class of polypeptides that can be used for membrane formation are elastin‐like polypeptides (ELPs).[^1b^, ^18^] The commonly used sequence motif (VPGXG)_*n*_ (shorthand notation: X_n_) is derived from tropoelastin, where X is any natural amino acid except proline and n is the number of pentapeptide repeats. Depending on the amino acid used for X the peptide displays different hydrophobicity.^[19]^

We have recently shown that ELPs can form ≈200 nm sized vesicular structures for the compartmentalization of biochemical reactions such as transcription of RNA aptamers and the expression of fluorescent proteins.^[16a]^ In the present work we demonstrate the fabrication of much larger, cell‐sized polymersomes using a solvent evaporation method.^[20]^ In contrast to the glass beads method previously used by Vogele et al.^[16a]^ the necessary peptide film was formed on the inner glass surface of a round‐bottom flask, which resulted in vesicle sizes in the μm scale (Figure 1). Importantly, ELP polymersomes encapsulating transcription (TX) or transcription‐translation (TX‐TL) reactions displayed a strong increase in size when they were externally supplied with additional membrane peptides. In several cases, fusion events between adjacent vesicles were observed as well.

**Figure 1:**
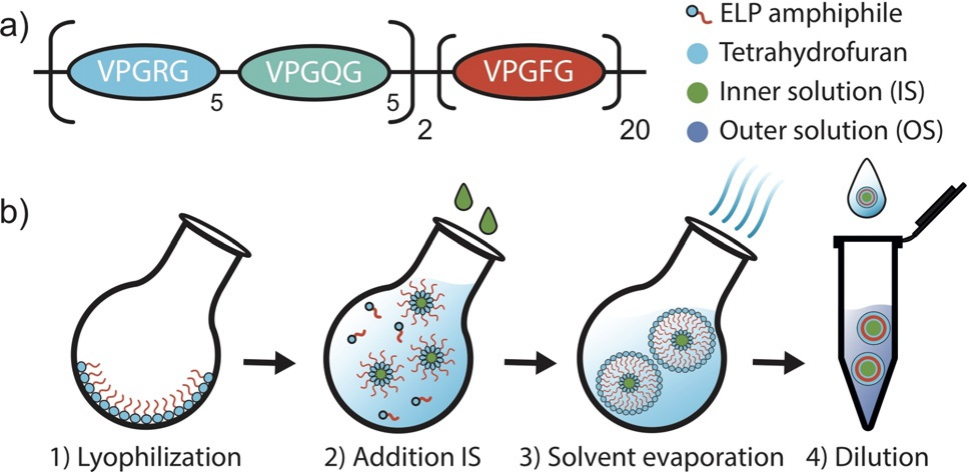
a) Scheme of the sequence design of the amphiphilic ELP. b) Graphical depiction of the workflow for the solvent evaporation method to produce giant ELP vesicles.

Transcription of RNA aptamers inside the vesicles resulted in mixed growth behaviors, in which some vesicles started to shrink at one point, while others continued to grow. By contrast, when expressing the fluorescent protein YPet, 97 % of the vesicles continually increased in size; for the remaining two vesicles no clear shrinkage or growth could be observed. Our experiments hence demonstrate biochemically driven growth of peptide‐based synthetic cellular structures, which could set the stage for competition and selection dynamics emerging among such compartments.

We used an ELP with the sequence (R_5_Q_5_)_2_‐F_20_ (in shorthand notation) as the amphiphilic membrane component (Figure 1, Supporting Information section 3). This sequence design ensures that the peptides contain a well‐structured hydrophobic tail and a random coil polar head group under our standard experimental conditions. Peptide expression and purification were performed as described previously.^[21]^

Controlled formation of giant peptide vesicles was carried out through solvent evaporation based on a protocol originally introduced by Marsden et al.[^16b^, ^20^] Initially, the amphiphilic ELPs were lyophilized in a round‐bottom flask to completely remove water from the peptides (Figure 1 b). The peptides were then re‐dissolved by the addition of tetrahydrofuran (THF) and sonication. For encapsulation, the internal solution (IS), which contained a defined amount of sucrose in purified water, was added to the THF/ELP mixture, followed by agitation and incubation at room temperature. Because of the amphiphilic nature of the ELPs used, droplets of the inner solution are formed, which are covered and stabilized by an ELP layer. Subsequent formation of a peptide double‐layer at the droplet interface, and hence the formation of vesicles, was accomplished by the removal of the THF solvent through evaporation. Nonetheless, residual solvent within the ELP double layer cannot be ruled out entirely.

After formation the vesicles were mixed with an outer solution (OS) to dilute residual IS. The OS contained an isotonic amount of glucose as well as a low percentage of Triton X‐100 (0.01 %). Due to the higher density of the IS, the vesicles sedimented at the bottom of a microscopy sample chamber and could thus be easily observed for several hours. If an osmotic shock is applied the vesicles vanish (Figures S5, S6).

The solvent evaporation method resulted in two populations of vesicles of different sizes, namely small vesicles (SV) with radii far below 1 μm and larger vesicles with sizes spanning two to three orders of magnitude, which we will collectively refer to as giant vesicles (GV). In order to characterize the size of the vesicles across these scales, we utilized transmission electron microscopy (TEM), dynamic light scattering (DLS) as well as light microscopy (LM). For the SVs, a mean radius of 0.03 μm ±0.01 μm was determined using TEM (Figure 2), where the estimated uncertainty is the standard deviation of the distribution. The GV fraction displayed a wide range of radii between 400 nm and several micrometers. Due to the size resolution limits of the DLS and LM characterization methods, we were not able to acquire a quantitative size distribution across the whole range, but they allowed us to determine the lower and upper size limits of the GV population (Figure 2 b). In LM, we occasionally also observed vesicles with radii much larger than 10 μm. A rationale for the occurrence of the SV and GV populations is the presence of two alternative processes of vesicle formation. SVs are presumably created through spontaneous formation in aqueous solution from ELP monomers, whereas the GVs are generated only through the application of the solvent evaporation method.

**Figure 2:**
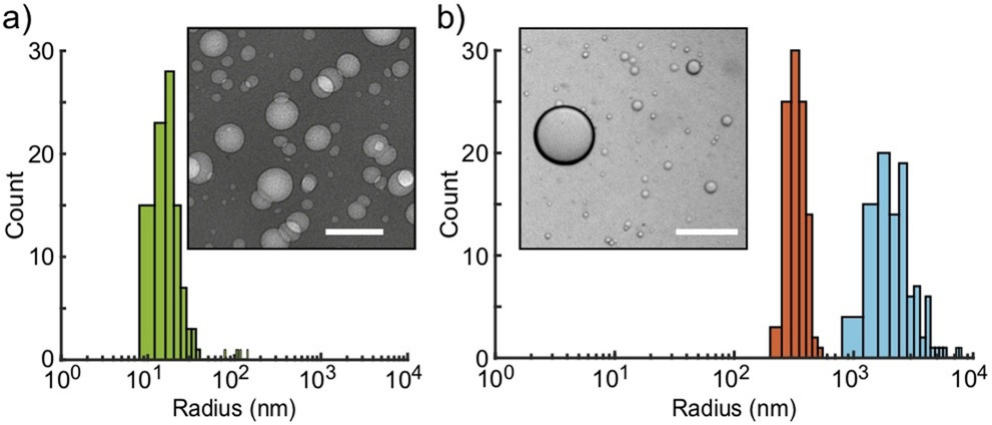
a) Size distribution of ELP vesicles analyzed with TEM. Sample size: N=100. Inset: TEM image of ELP vesicles. Scale bar: 100 nm. b) Size distributions of vesicles using DLS (orange) and LM (cyan). Sample sizes: N=100. Inset: LM image of ELP vesicles. Scale bar: 50 μm.

We next studied the capability of the GVs for fusion and growth. We speculated that—similarly as previously observed with fatty acid vesicles^[4]^—an osmotic imbalance could lead to an influx of water and thus promote vesicle growth. In our experiments the imbalance was created through biopolymerization of polyelectrolytes. We therefore transcribed the fluorogenic RNA aptamer dBroccoli inside the vesicles by encapsulating a transcription mix containing T7 RNA polymerase, rNTPs, template DNA, the ligand of the aptamer, DFHBI (3,5‐difluoro‐4‐hydroxybenzylidine imidazoline), and sucrose (Supporting Information section 1.2). In order to be able to control the start of the transcription reaction, we separately produced two types of vesicles, which were either missing the template DNA or the T7 RNA polymerase (Figure 3 a). In the experiments, these vesicles were mixed, resulting in the transcription of fluorescent aptamers only inside of vesicles, which were generated through fusion of the two vesicle types. We added DNase I to the OS to prevent transcription by accidentally released transcription mix. In addition, the surrounding OS was supplemented with a low percentage of Triton X‐100 (0.01 %) and an isotonic amount of glucose to balance the initial osmotic pressure in the vesicles. Control experiments showed that no TX activity was observed in the absence of Triton X‐100, which apparently promoted vesicle fusion. Surprisingly, Triton X‐100 alone cannot induce peptide vesicle fusion (Figure S4), and we suppose that a slight osmotic imbalance is necessary for successful fusion events. It was essential to add additional ELP monomers (200 μm) to the OS to facilitate vesicle growth through their incorporation into the membrane. We found that in the absence of external ELPs, the observed vesicles were generally smaller and less abundant. Furthermore, the vesicles were not stable during the experiments and tended to shrink.

**Figure 3:**
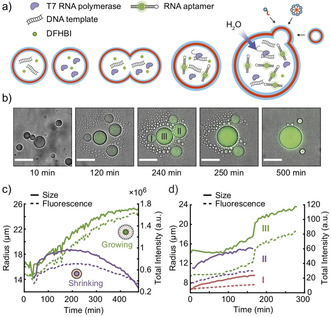
a) Schematic illustration of vesicle fusion between vesicles containing complementary components of a transcription mix, which is used to start the synthesis of dBroccoli RNA aptamers in situ. b) Time series of LM images (overlay of bright field and fluorescence) of growing ELP vesicles. Scale bars: 40 μm. c) Typical time traces for vesicle radius (solid lines) and fluorescence intensity (dashed lines) of continuously growing vesicles (green) and growth followed by shrinkage (purple). d) Exemplary time traces for vesicle radius (solid) and fluorescence (dashed) of fusing vesicles (I–III) indicated in b). After the fusion of II and III, the combined (II+III) fuse with I.

The initial vesicle population was highly polydisperse in size, which likely resulted in large variations in the contents of the compartments generated via fusion of the two types of vesicles. This in turn was expected to result in a broad distribution of transcriptional activities among the vesicles.^[22]^ As shown in Figure 3 b and Video S1, vesicles containing the transcription mix strongly varied in number and size over a time period of several hours. Next to a strong increase in size of the GVs, the appearance of small “satellite” vesicles around the GVs was observed. These are potentially generated by spontaneous budding events,^[23]^ membrane instabilities followed by budding due to osmotic imbalances and Triton X‐100 or interactions with the microscopy glass slide. However, similar vesicles were observed to emerge throughout the whole micrograph, which suggests that they could also simply originate from growing SVs, whose size initially was below the observation limit.

The observed growth of the vesicles is consistent with our expectation that compartmentalized RNA polymerization is accompanied by an increasing osmotic pressure in the vesicles.^[4]^ In the absence of bio‐polymerization reactions no vesicle growth was observed, even with externally provided ELPs (Figure S4, Video S2). Figure 3 c shows example time traces for growing vesicles and for growth followed by shrinkage when RNA aptamers are transcribed inside a vesicle. When in close proximity, two or more GVs can also fuse and thereby rapidly increase in size. We find that in all fusion events, the final intensities, radii and volumes are slightly less than expected from the sum of the fusing vesicles (Figures 3 d, S8, Video S3), implying that some of the vesicle content leaks out during fusion events. The excess membrane resulting from fusion may either be lost to solution, or incorporated into a multilamellar membrane structure.

In order to understand the dynamics of vesicle growth and shrinkage, we have to consider the interplay of compartmentalized RNA polymerization, the incorporation of externally provided ELPs into the membrane and water influx. As shown in Figure 4 a, fluorescence intensities and volumes are almost linearly correlated, which indicates that the concentration of the transcribed RNA molecules in the vesicles stays approximately constant. This in turn suggests that water influx into the growing vesicles is fast enough to compensate for the excess osmotic pressure generated by the newly formed polyelectrolytes.[^4^, ^24^] As can be seen in the inset of Figure 4 a, the global linear trend is occasionally interrupted by phases of alternating growth and shrinkage, where the correlation between intensity and volume becomes non‐linear.

**Figure 4:**
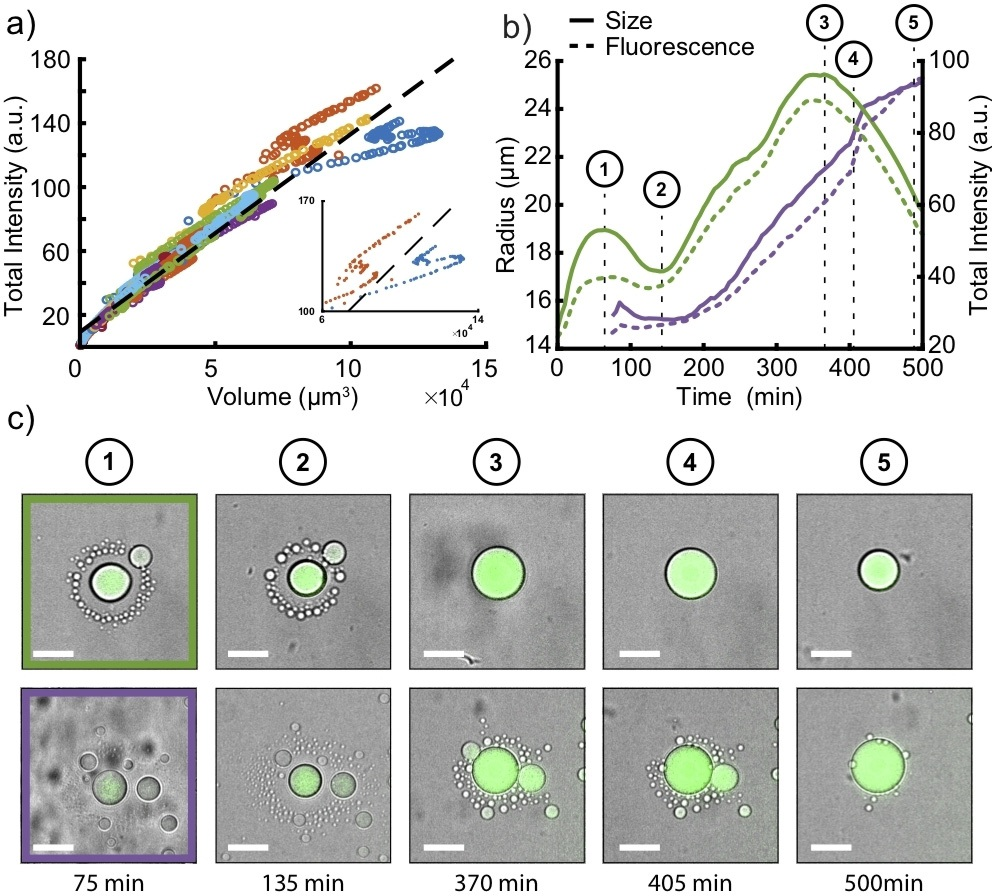
a) Representative plots of total vesicle fluorescence intensity vs. vesicle volume over a time course of 500 min. The different colors indicate different vesicles (N=20). The dashed line shows a linear fit to all 20 vesicles (see Supporting Information). Inset: intensity vs. volume of two exemplary vesicles showing growth, shrinkage and growth one after another. b) Typical time traces for vesicle radius (solid lines) and fluorescence intensity (dashed lines) of a growing vesicle (purple) and a vesicle showing alternating growth and shrinking phases (green). For all curves a 4‐point moving average filter was used. Numbers 1–5 indicate the corresponding LM images. c) Time series of LM images (overlay of bright field and fluorescence). The green and purple frame indicate the corresponding trace in b) Please note that data analysis started after *t*=75 min due to ongoing sedimentation. The corresponding video S4.2 starts at *t*=0 min. Scale bars: 40 μm.

Our experimental observations further suggest that the availability of a supply of ELPs (as monomers, micelles or small vesicles) in their immediate vicinity determines whether vesicles will grow or shrink. Some vesicles are found to repeatedly grow and shrink, and finally even disappear (Figure S8, Videos S4.1 and S4.2). Figures 4 b,c show two representative examples for vesicles surrounded by either many or by a few smaller vesicles. It can be clearly seen that in both cases the smaller vesicles slowly disappear whereas the largest vesicle continually grows. We suppose that the large sizes of the central vesicles are reached by the consumption of the surrounding “prey” vesicles. As soon as the supply of prey vesicles is depleted, the central vesicle starts to shrink (Figure 4 b, green).

In another set of experiments, we encapsulated a bacterial cell extract‐based protein expression system (TX‐TL) mixed with sucrose solution and a plasmid encoding the yellow fluorescent protein YPet into the GVs (Supporting Information section 1.2). The OS contained glucose, ELPs, and kanamycin to inhibit translation outside of the GVs by potentially present non‐encapsulated TX‐TL components. The vesicle fluorescence (Figures 5 a,b; S11) and the corresponding Video S5 show a similar behavior as for vesicles containing only a transcription reaction (Figures 3 c,b; S8, S11). In contrast to the TX mixture, TX‐TL had to be prepared already before encapsulation, resulting in YPet synthesis prior to the measurement. Bulk measurements in a fluorescent plate reader using the same TX‐TL protocol show an increase in fluorescence after roughly 20 min (Figure S13), whereas the time delay between preparation and microscopic observation of the vesicles lasted up to 45 min.

**Figure 5:**
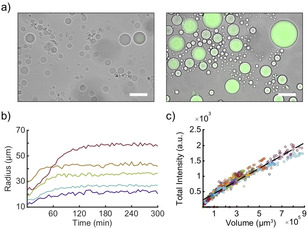
a) LM images (overlay of bright field and fluorescence) of vesicles containing TX‐TL after 15 min (left) and 24 h (right). The fluorescence is produced by YPet. Scale bars: 50 μm. b) Typical radius vs. time traces of vesicles expressing YPet. **c)** Fluorescence intensity vs. volume of 77 vesicles expressing YPet over 3 h. The dashed line shows a linear fit to all 77 vesicles.

As in the TX case the correlation between YPet fluorescence and volume is approximately linear (Figure 5 c), which again suggests a balance between *in vesiculo* production of biopolymers and osmotically driven growth. In contrast to the TX mix, the much more complex cell extract contains nearly the complete proteome of *BL21 rosetta E. coli* cell, in which case the osmotic pressure of the vesicle will be influenced by a more complex network of biochemical reactions. Only very few cases of shrinking vesicles were observed, but most vesicles enter a plateau phase of constant volume and constant YPet fluorescence after completion of the TX‐TL reaction, which indicates that an osmotic equilibrium has been attained between the inner and the outer solution.

In conclusion we have demonstrated the generation of cell‐sized peptide vesicles, which upon encapsulation of cell‐free transcription and protein expression reactions exhibit volume changes over at least an order of magnitude in several cases. The size changes appear to be caused by a combination of fusion events and osmotically driven growth, when fed with membrane components from the outside. Vesicle growth promoted by internal bio‐polymerization reactions is thus much more pronounced than previously observed for other membrane systems, which may be related to the high permeability of the ELP membranes for water combined with their considerable mechanical stability.

It is conceivable that usage of more complex membrane compositions will facilitate the implementation of other cell‐like behaviors such as compartmental division and reproduction. In fact, we already observed occasional budding events in our experiments (Supporting Information, Figure S10, Video S6), which may be taken as precursors for such processes. As the growth of our peptide vesicles is coupled to internal gene expression activity, our results also may lay the ground for a competition between different peptide compartments based on the efficiency of the compartmentalized reactions.

## Conflict of interest

The authors declare no conflict of interest.

## Supporting information

As a service to our authors and readers, this journal provides supporting information supplied by the authors. Such materials are peer reviewed and may be re‐organized for online delivery, but are not copy‐edited or typeset. Technical support issues arising from supporting information (other than missing files) should be addressed to the authors.

Supplementary
