# Supplementary material for: Growth of Giant Peptide Vesicles Driven by Compartmentalized Transcription–Translation Activity
Source: Chemistry. 2020 Nov 24;26(72):17356–60. doi: 10.1002/chem.202003366 (PMC7839564; doi:10.1002/chem.202003366)
Supplement: Supplementary file 1 — Supplementary [file CHEM-26-17356-s001.zip › Frank_Supporting_information_accepted.pdf]

Table of Contents

1 Materials and Methods . . . 3

1.1 Materials . . . 3

1.2 Methods . . . 3
1.2.1 Expression and purification of elastin-like peptides . . . 3
1.2.2 Solvent evaporation method . . . 3
1.2.3 SDS-Polyacrylamide Gel Electrophoresis . . . 4
1.2.4 Transcription reaction (TX) . . . 4
1.2.5 Transcription translation reaction (TX-TL) . . . 4
1.2.6 Transmission Electron Microscopy (TEM) . . . 5
1.2.7 Dynamic Light Scattering (DLS) . . . 5
1.2.8 Light Microscopy (LM) . . . 5
1.2.9 Fluorescence plate reader measurements . . . 6

2 Supporting Data . . . 7

2.1 SDS-PAGE . . . 7
2.2 Vesicle Size . . . 7
2.3 Osmotic stress experiment . . . 8
2.4 RNA aptamer fluorescence . . . 8
2.5 Vesicle growth through RNA aptamer transcription . . . 8
2.6 *In vesiculo* expression of fluorescent protein YPet . . . 9
2.7 Light microscopy (LM) . . . 11
2.8 Individual Budding/fission events . . . 11

3 Nucleic Acid Sequences . . . 12

4 References . . . 13

1 Materials and Methods

1.1 Materials

Chemicals were ordered from Sigma Aldrich or Carl Roth, if not otherwise mentioned.

1.2 Methods

1.2.1 Expression and purification of elastin-like peptides

The ELP variants were constructed via Golden Gate cloning and inserted into the expression vector pET28b(+). The plasmid was transformed into *BL21 DE3 pLys E.coli* cells (Promega) via heat shock. The correctness of the plasmid was confirmed by Sanger sequencing. An overnight culture of 5 ml was used to inoculate 750 mL LB medium containing 100 µg/mL of kanamycin and chloramphenicol (Kan/Cm). For the incubation at 37 °C in a shaking flask an antifoam agent Antifoam 204 (other company than Sigma?) was added. The cells were grown up to an optical density of 0.6 - 0.8 before induction by 1 mM Isopropyl ß-d-1-thiogalactopyranoside (IPTG). Purification was performed following the protocol established by McEwan et al. [1]. After incubation at 16 °C for about 16 h the cells were pelleted at 4000 rcf at 4°C for 15 min, followed by washing cold with 30 mL of 1x PBS buffer and centrifuging at 7100 rcf at 4 °C for 10 min. The cell pellet was lysed by the addition of a lysis buffer consisting of 1x PBS, 1 mg/mL lysozyme (from chicken egg white), 1 U/mL Turbo DNase (Ambion), 1 mM Benzamidine and 1 mM Phenylmethylsulfonyl flouride (PMSF). The crude lysate was aliquoted in 2 mL reaction tubes and incubated at 65 °C for 10 min before being centrifuged at 16000 rcf at 4 °C for 10 min. The collected supernatant was mixed with 3 M NaCl and heated at roughly 75 °C until the sample turned cloudy. The sample was centrifuged at 7100 rcf at room temperature for 15 min. Afterwards the pelleted ELPs were resuspended in H_2O_{dd} (4 °C) and incubated at 4 °C for 1h, followed by centrifugation at 7100 rcf at 4 °C for 15 min. These purification steps were repeated 3 - 4 times, and finally the samples were dialyzed in a Slide-A-Lyzer Dialysis Cassette (Thermo Fisher Scientific) with a 10 kDa cut-off at 4 °C overnight. The purified samples were analyzed using SDS-PAGE.

1.2.2 Solvent evaporation method (adjusted protocol from ref. [2])

After purification, the intended amount of ELPs dissolved in H_2O_{dd} was pipetted into a 25 mL round flask. For our experiments a concentration of 290 µM ELPs was used. The ELP solution in the flask was frozen using liquid nitrogen and quickly placed into a desiccator at room temperature. The peptides were lyophilized for roughly 1 h until all liquid was evaporated. Afterwards the peptides were dissolved using 1 ml of dry THF and were subsequently sonicated in a water bath at room temperature for 30 min (90W, 37 kHz; Elmasonic S10H). The sample which was intended to be encapsulated was completely pipetted to the ELP/THF solution, vortexed for 5 – 10 s, followed by an incubation at room temperature for 15 min. For size measurements and TX experiments 60 µL of inner solution (IS) was added, and for TXTL experiments 80 µL of IS was added. Afterwards, the residual liquids were removed using a rotary evaporator (Heidolph GmbH & CO.KG, Germany) at room temperature with an inner pressure of about 100 mbar for 5 – 10 min. Evaporation was stopped as soon as a higher viscosity of the sample is observed as well as the two distinct phases are visibly mixed. The vesicles were then added to an outer solution in a 1:2 ratio and mixed by pipetting. The solution was then put into the make-shift observation chambers (Figure S13) and analyzed under an Olympus IX71 (Olympus, Japan) microscopy setup.

Depending on the experiment, the measurements were conducted for several hours at room temperature or were heated to the desired temperature.

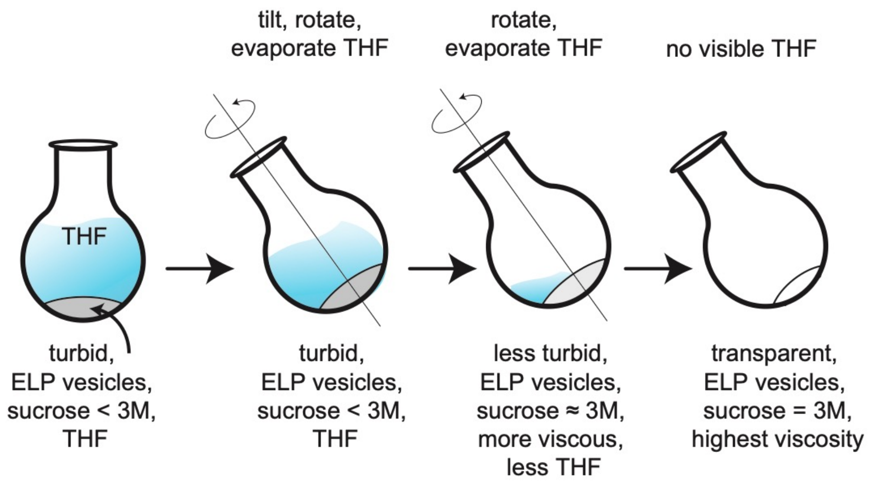

Figure S1. Graphical illustration of vesicle formation using the solvent evaporation method. After the addition of the inner solution (IS) including 3 M sucrose vesicles are formed which settle at the flask bottom. The grey area depicts a viscous aqueous phase containing vesicles, residual IS and residual THF. During THF evaporation this viscous phase loses residual THF and gets transparent. At this point there should be almost no THF left and the sucrose concentration should have reached 3M. Evaporation gets stopped here.

1.2.3 SDS-Polyacrylamide Gel Electrophoresis (SDS-PAGE)

The ELP expression and purification rates were carried out using 12.5% SDS-PAGE gels. For the separating gel 12.5% of acrylamide-bisacrylamide (37.5:1) solution, 380 mM of Tris (pH 8.8), 0.1 % SDS, 0.1 % APS and 0.04% TEMED were mixed in a 50 mL tube. The right concentrations were adjusted by addition of H_2O_{dd} before pipetting the solution into the Mini Gel. For the stacking gel 1% of acrylamide-bisacrylamide (37.5:1) solution, 38 mM Tris (pH 6.6), 0.02% SDS, 0.02 % APS and 0.02 % TEMED were mixed in a 50 mL tube. The stacking gel mix was pipetted into the gel cassette and the comb was inserted into the stacking gel. Before the samples were loaded into the gel, they were prepared with 2x Laemmli buffer and were heated at 85 °C for 10 min. Because of the phenylalanine-rich hydrophobic domain of the purified ELPs, commercial Coomassie stain was used to stain the gel for about 20 min. Images were made using a Quantum CX5 gel documentation system (Vilber Lourmat, Marne-la-Vallée).

1.2.4 Transcription reaction (TX)

The main components for this reaction were 4 U/µL T7 RNA polymerase, 200 nM of the DNA template encoding the dBroccoli aptamer, 10 µM DFHBI and a buffer solution which supports the transcription process. This buffer solution consisted of the provided 10x RNAPol reaction buffer and 16 mM (4 mM each) ribonucleoside tri-phosphates (rNTPs) (NEB, USA). Additionally, the concentrations of 125 mM KCl and 15 mM $MgCl_2$ were adjusted to optimize the performance of the T7 RNA polymerase as well as the fluorescence of the RNA aptamer. To ensure that the polymerization reaction was not started prematurely, the DNA template and the T7 RNA polymerase were encapsulated separately. As an additional measure against RNA degradation, 0.5 U/µL murine RNase inhibitors (NEB, USA) were added to the solution. For a better observation over a longer time period, a density gradient between the inner and outer solution was used to reduce the movement of the vesicles. Therefore, the encapsulated

solution contained 3 M sucrose while the outer solution contained 3 M of glucose. Because of the different specific densities of the sugars, the vesicles sedimented in the observation chamber. The outer solution also contained 200 U/mL DNase I (NEB, USA) to suppress a background signal by degrading non-encapsulated DNA templates. After preliminary testing, 0.01% (v/v) Triton X-100 was included in the outer solution to reduce the amount of unspecific aggregates as well as to induce fusion of the vesicles [3]. The observation under the microscope was performed for several hours at room temperature.

1.2.5 Transcription translation reaction (TX-TL)

Cell extract was produced according to the protocol provided by Sun et al. [4] and stored at -80 °C after freezing aliquots in liquid nitrogen. The prepared *BL21-Rosetta 2 (DE3)* cell extract and buffer solution were thawed on ice. For the encapsulation of the TX/TL mix, 28.5 µL cell extract, 35.7 µL buffer solution, 5 nM phenol-chloroform purified DNA plasmid were mixed and brought to a volume of 80 µL by the addition of 3 M sucrose; the buffer composition can be seen in table S1. The components were kept on ice during pipetting to minimize enzymatic activity. The following encapsulation process was carried out using solvent evaporation. Background signals were suppressed by the addition of kanamycin (50 µg/mL) to the isotonic outer solution containing glucose. Imaging was carried out at 29°C using a microscope equipped with an incubator for several hours.

Table S1. Composition of TXTL buffer

Components	$C_{solution}$
Hepes (pH 8)	50 mM
ATP, GTP	1.5 mM
CTP, UTP	0.9 mM
tRNA	0.2 mg/mL
Coenzyme A	0.26 mM
NAD	0.33 mM
Folinic acid	0.75 mM
Spermidine	68 µM
cAMP	1 mM
PEP	30 mM
DTT	1 mM
PEG-8000	2%

1.2.6 Transmission Electron Microscopy (TEM)

The freshly prepared solution with vesicles was adsorbed on glow-discharged formvar-supported carbon-coated Cu400 TEM grids (FCF400-CU, Science Services, Munich, Germany) for 2 min. Next the

vesicles were negatively stained using a 2% aqueous uranyl formate solution with 25 mM sodium hydroxide for 45 s. Finally, the grid was dried and stored in vacuum for 30 min. For imaging a Philips CM100 transmission electron microscope at 100 kV was used. For acquiring images an AMT 4 megapixel CCD camera was used and imaging was performed at magnification between x 8900 and × 15,500. Image processing was carried out using the plugin Scale Bar Tools for Microscopes for Java-based software Fiji. All measurements were analyzed using the processing software MATLAB (MathWorks Inc., USA).

1.2.7 Dynamic Light Scattering (DLS)

Dynamic light scattering was carried out using the DynaPro Nanostar (Wyatt technology corporation, Santa Barbara). The vesicles were prepared by the solvent evaporation method, where the inner solution was H_2O_{dd}. For one distribution a set of 100 single measurements where performed for 2 s and averaged afterwards. The values were averaged and processed with the DYNAMICS software using a CONTIN-like algorithm.

1.2.8 Light Microscopy (LM)

The long time measurement was performed by using self-made observation chambers (SI Figure S14). A rubber O-ring was fixed to a thin hydrophobically covered glass slide using a two component adhesive. The samples are mixed and pipetted into the O-ring until they fill the whole volume of the ring. To prevent excessive evaporation another cover slide is slid on the O-ring from the side to prevent formation of air bubbles within the observation chamber. The chamber then is placed on top of a microscope slide (24 mm x 75 mm x 1.5 mm) and fixed with a thin piece of adhesive tape.
The observation of the vesicles was performed using an Olympus IX71 inverted microscopy setup with a motorized stage (Prior, UK). The vesicles were identified and observed in different regions of interest for different timespans ranging between 6-12 h. All measurements were conducted under 10x magnification. The individual frames were taken in 5 min intervals and depending on the experiment the exposure time for fluorescence microscopy was adjusted. Acquisition of measured values was performed using an automated droplet tracking algorithm for MATLAB [5]. Size as well as fluorescent intensity were determined in this manner.

1.2.9 Fluorescence plate reader measurements

Fluorescence measurements (cell-free expression and transcription) were carried out using the BMG FLUOstar Optima plate reader with the corresponding filter sets. The samples were processed using 20 µL reaction volumes in 384-well plates (Brand GmbH & CO.KG, Germany).

2 Supporting Data

2.1 SDS-PAGE

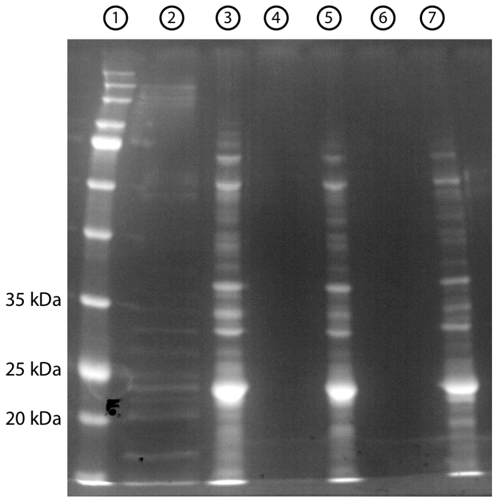

Figure S2. SDS gel of purified ELPs during ITC. Lane 1: prestained protein marker RotiMark TRICOLOR. Lane 2: Supernatant after first hot spin. Lane 3: Redissolved pellet after first cold spin. Lane 4: Supernatant after second hot spin. Lane 5: Redissolved pellet after second cold spin. Lane 6: Supernatant after third hot spin. Lane 7: Redissolved pellet after third cold spin.

2.2 Vesicle Size

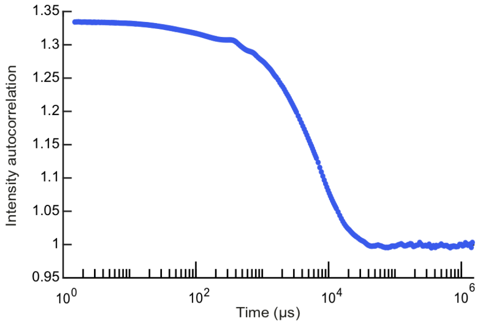

Figure S3. Typical intensity autocorrelation function from a DLS measurement.

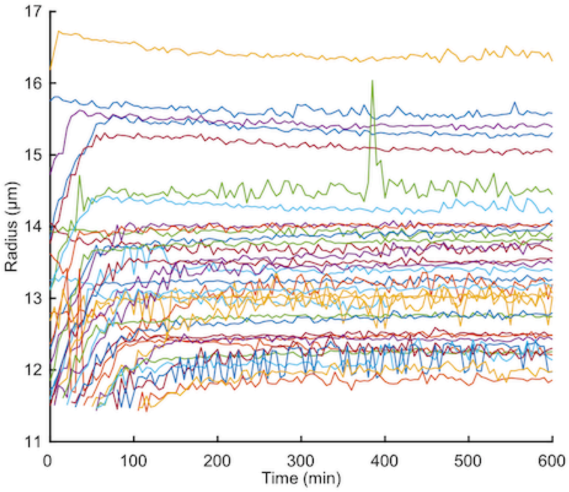

Figure S4. Vesicle radius over a time period of 10 h. Sample size N=38.

As a control we also observed ELP vesicles containing only 3 M sucrose in water, whereas the outer solution contained only 3 M glucose in solution; there were ELPs provided in the outer solution. Figure S4 shows the vesicle radius vs. time traces of 38 vesicles. The apparent initial increase in size can be explained through the on-going sedimentation of the vesicles. The latter can also be observed in long-term measurements (Supporting Video S1).
The automated droplet tracking algorithm covers only a small area of the vesicle outside of the focal plane, which grows when the vesicles sediment into the focal plane. Looking at vesicles with a larger radius, the opposite effect resulting from the same behavior is observable. After about 100 min most of the vesicles have a constant size after they settled on the surface of the glass slide.

2.3 Osmotic stress experiment

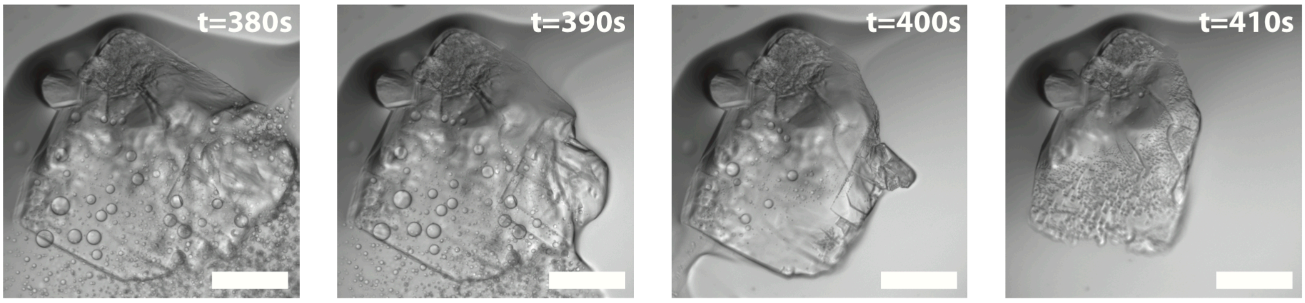

Figure S5. Microscopy time series of ELP vesicles containing only 3M sucrose and experiencing hypotonic shock. The vesicles are in close proximity to a sucrose crystal and the surrounding solution is saturated with sucrose. At time t=0 pure water was added the measurement chamber. At t=380 water is appearing from the right and diluting the sucrose environment. The vesicles disappear due to a high internal pressure (3 M sucrose). Scale bars: 200 µm.

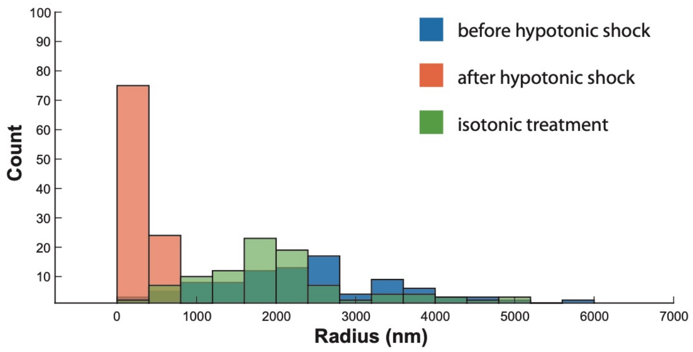

Figure S6. DLS measurement of ELP vesicle before and after a hypotonic shock. ELP vesicles filled with 1.5 M sucrose before the shock show a mean radius of 2,830 nm (blue), the same sample after the shock showed only a mean radius of 338 nm (orange). We assume after destruction due to the hypotonic shock single ELPs and membrane residuals spontaneously form smaller vesicles or aggregates. For a negative control 1.5 M sucrose was added to the ELP vesicles and the radius resulted in 2,185 nm (green).

2.4 RNA aptamer fluorescence

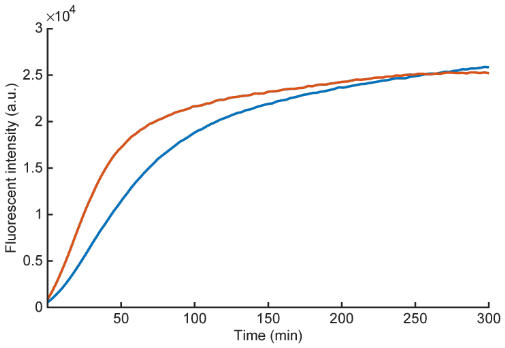

Figure S7. Duplicate plate reader measurement of TX reaction mix used.

The TX reaction mix used for encapsulation was measured in bulk as well to estimate the active time of transcription. Figure S7 shows two time-traces of the measured aptamer fluorescence in bulk. The samples were measured with excitation using a filter 485 +/-6 nm and an emission filter of 520 +/-17 nm.

2.5 Vesicle growth through RNA aptamer transcription

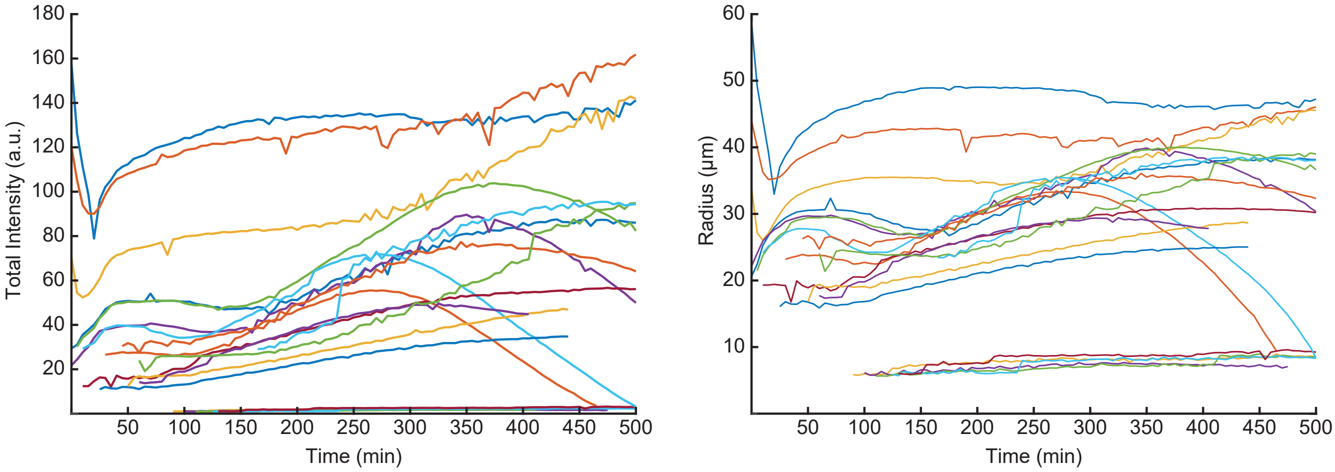

Figure S8. Measurement of fluorescence (left) and radius (right) of TX mix encapsulated in ELP vesicles. Color coding indicates the same vesicle. Sample size N=20.

Figure S8 shows the fluorescence intensities and the radii of 20 vesicles containing an active RNA polymerization process. ELP amphiphiles were provided in the outer solution. For excitation an LED lamp and excitation at 470 +/-20 nm were used, whereas emission was observed at 531 +/-13 nm. Depending on the observed vesicle a stationary or decreasing trend can be seen in fluorescent intensity and vesicle size. Very small vesicles only show small changes in fluorescence and size within the observation time. Depending on the observed vesicle, initial increase in size and fluorescence can either

be seen continuing until the end of the measurement or individual vesicles start to shrink after a certain time.

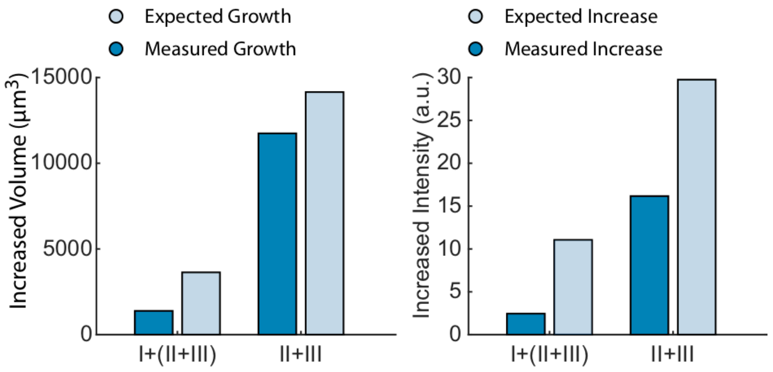

Figure S9. Bar plot comparing measured (dark blue) and expected (light blue) values after fusion events observed during long-term TX measurements. Bars represent the volume resp. the fluorescence difference between the values after and before fusion. Abscissa numbering refers to vesicles depicted in Fig. 4b.

Measurement of fused vesicles has shown that neither the expected volume nor the fluorescent intensity adds up after fusion of the observed vesicles. The expected values (light blue) surpass the measured values (dark blue) in both cases of the observed fusion events. Similar values were measured for several more fusion events.

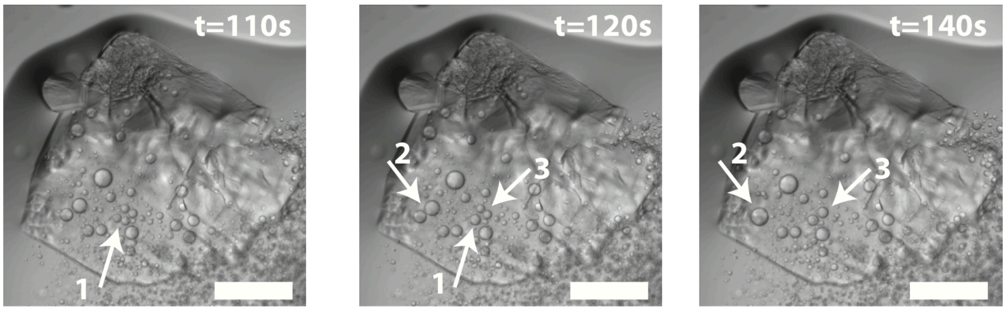

Figure S10. Microscopy time series of ELP vesicles containing 3 M sucrose, but no Triton X-100. The vesicles are in close proximity to a sucrose crystal and the surrounding solution is saturated with sucrose. White arrows and numbers indicate fusion events. Scale bars: 200 µm.

2.6 *In vesiculo* expression of fluorescent protein YPet

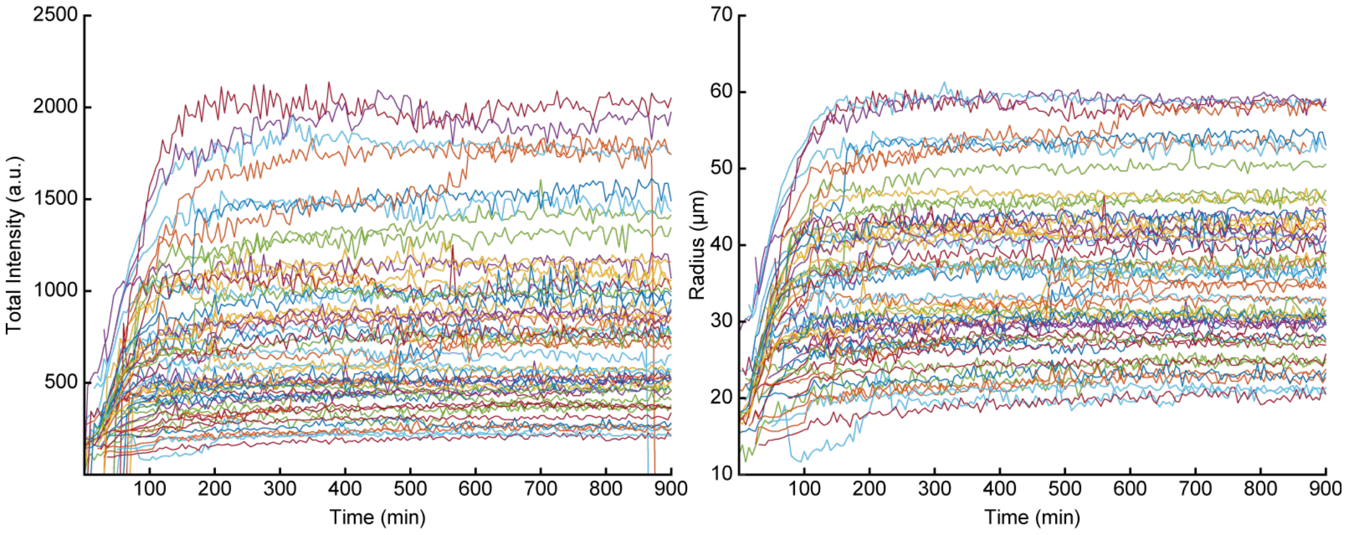

Figure S11. Increasing fluorescent intensity (left) and vesicles radius (right) for 900 min. Color coding indicates the same vesicle. Sample size N=77.

Vesicles encapsulating an in vitro transcription translation reaction were measured at 29 °C for 900 min. ELP amphiphiles were provided in the outer solution. For excitation an LED lamp and an excitation filter (470 +/-20 nm) were used, whereas emission was observed 531 +/-13 nm. All vesicles show an initial increase in size as well as in fluorescent intensity up to roughly 160 min after the measurement was started. Until the end of the measurement all vesicles show no further change in size and fluorescence.

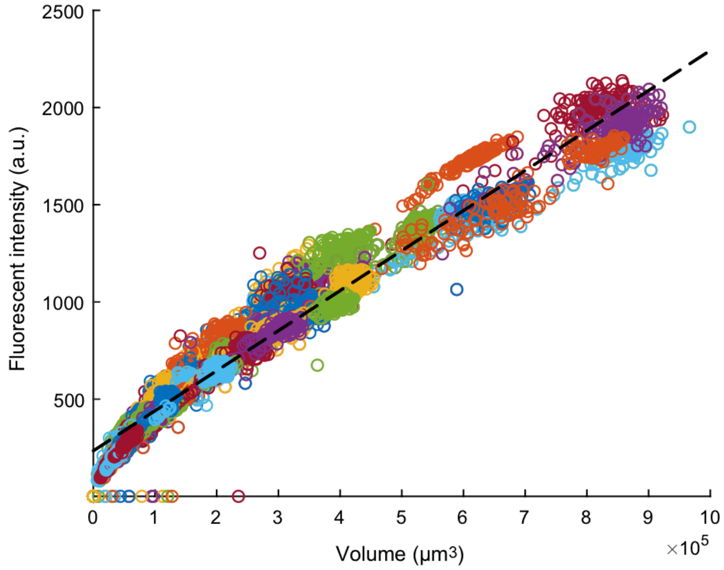

Figure S12. Scatter plot of fluorescent intensity vs. vesicle volume. Individual vesicles measured over a time period of 900 min. Each color represents an individual vesicle. Sample size N=77.

The dashed line is a linear fit to all values of the 77 observed vesicles. On a first approximation fluorescence intensity and volume are linearly correlated. Some vesicles appear in clusters, which can be explained by nearly constant values in fluorescence and volume after about 160 min.

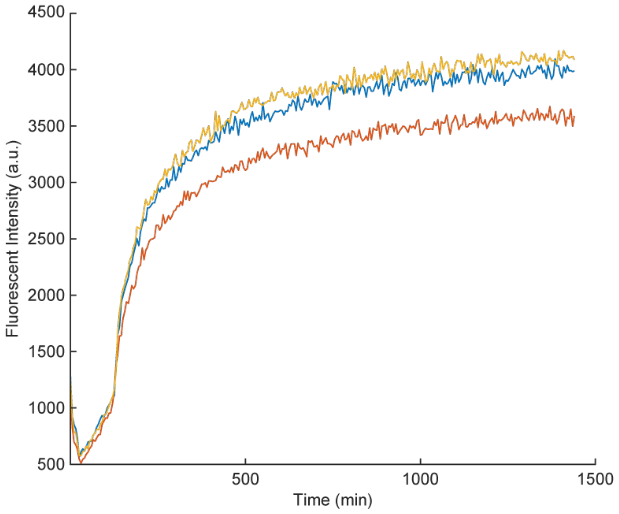

Figure S13. Measurement of YPet synthesis in a plate reader for 24 h. The shown curves represent triplicate measurement with identical composition.

The synthesis activity of the encapsulated transcription translation reaction was measured using fluorescence plate reader measurements. Figure S13 shows a biological triplicate at 37 °C for 24h. The initial decrease in fluorescence can be explained by the consumption of NADH which shows auto fluorescence within the observed wavelengths. For excitation a filter of 497 +/-15 nm was used, whereas emission was observed at 540 +/-20 nm.

2.7 Light microscopy (LM)

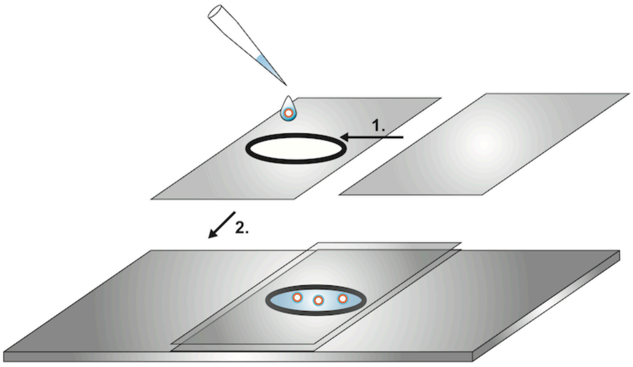

Figure S14. Schematic drawing of self-made observation chamber for long-term microscopy measurements.

The formed vesicles were mixed with the outer solution (OS) and pipetted into an O-ring fixated on the surface of the glass slide. The second cover glass is slid on top from the side to prevent air bubbles within the observation chamber (1). The sealed chamber is put on top of an observation glass slide and fixated with adhesive tape (2).

2.8 Individual budding/fission events

During a single TX measurement it was possible to observe multiple budding/division events of a single ELP vesicle (see Fig. S15 and Video S6). A possible explanation could be an insufficient mixing of the formed vesicles and the surrounding OS, resulting in a locally increased concentration of Triton X-100. The latter is known as agent enforcing restructuring of vesicle membranes [3b]. The white errors in the

central and right image depict spontaneous deformations of the membrane which is well-known for budding events of lipid vesicles [6]. These deformations lead to multiple budding/division events indicated by black arrows. Immediately after budding the daughter cells fuse.

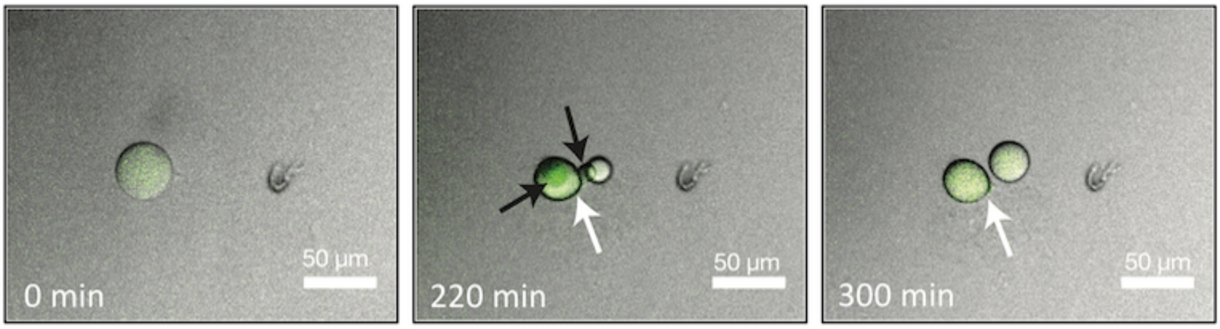

Figure S15. First budding/division events. The ELP vesicle contains transcription mix to transcribe the fluorescent RNA aptamer dBroccoli.

3 Nucleic Acid Sequences

Gene sequence $(R_5Q_5)_2F_{20}$

ATGGTACCTGGCCGGGGGGTTCCCGGACGAGGAGTACCCGGGCGGGGAGTGCCAGGACGCGGCGTTCCTGGGCGAG
GTGTGCCTGGTCAAGGAGTTCCTGGACAGGGAGTACCAGGACAAGGTGTCCCTGGTCAGGGTGTTCCAGGCCAAGG
AGTACCTGGCCGGGGGGTTCCCGGACGAGGAGTACCCGGGCGGGGAGTGCCAGGACGCGGCGTTCCTGGGCGAGGT
GTGCCTGGTCAAGGAGTTCCTGGACAGGGAGTACCAGGACAAGGTGTCCCTGGTCAGGGTGTTCCAGGCCAAGGAG
TGGGTGTTCCGGGCTTTGGTGTCCCAGGTTTCGGCGTACCGGGCTTTGGTGTTCCTGGTTTCGGCGTGCCGGGCGT
GGGTGTTCCGGGCTTTGGTGTCCCAGGTTTCGGCGTACCGGGCTTTGGTGTTCCTGGTTTCGGCGTGCCGGGCGTG
GGTGTTCCGGGCTTTGGTGTCCCAGGTTTCGGCGTACCGGGCTTTGGTGTTCCTGGTTTCGGCGTGCCGGGCGTGG
GTGTTCCGGGCTTTGGTGTCCCAGGTTTCGGCGTACCGGGCTTTGGTGTTCCTGGTTTCGGCGTGCCGGGCTGGCC
GTGATAAC

Gene sequence YPet

ATGTCTAAAGGTGAAGAACTGTTTACGGGTGTCGTGCCGATTCTGGTCGAGTTGGACGGCGACGTGAACGGT
CACAAATTCAGCGTGAGCGGCGAGGGCGAGGGTGACGCGACGTACGGTAAGCTGACTCTGAAGCTGCTGTGC
ACCACGGGTAAATTGCCGGTTCCGTGGCCGACCCTGGTCACGACGCTGGGTTATGGTGTACAATGTTTTGCA
CGCTATCCGGACCACATGAAACAGCACGATTTCTTCAAGAGCGCGATGCCGGAAGGCTATGTTCAGGAACGT
ACCATCTTTTTCAAAGATGATGGTAATTACAAAACCCGCGCAGAAGTGAAGTTCGAGGGTGACACCCTGGTG
AACCGTATTGAGCTGAAGGGTATTGACTTCAAGGAAGATGGCAATATTCTGGGTCACAAACTGGAGTACAAC
TATAACAGCCATAACGTCTACATCACCGCGGATAAGCAAAAAAATGGTATCAAAGCAAATTTCAAGATTCGC
CACAACATCGAAGATGGCGGCGTGCAACTGGCCGATCATTATCAGCAGAATACCCCAATCGGTGACGGTCCG
GTGCTGTTGCCGGATAACCACTACCTGAGCTATCAAAGCGCGTTGTTCAAAGACCCGAATGAAAAACGTGAC
CACATGGTTCTGCTGGAATTTCTGACCGCTGCGGGCATCACTGAAGGCATGAATGAACTGTACAAGACGCGT
GGTGGCGGCGGTTCGATGAGCAAGACTATCGTTTTGTCCGTCGGCGAGGCTACCCGTACCTTGACCGAAATT
CAATCCACCGCGGACCGTCAAATTTTTGAGGAAAAAGTCGGTCCTCTGGTGGGTCGTCTGCGTCTGACCGCG
AGCCTGCGCCAGAACGGTGCCAAAACGGCATACCGTGTTAATCTGAAACTGGATCAGGCCGACGTTGTGGAC
AGCGGTCTGCCGAAAGTCCGCTACACCCAGGTGTGGAGCCACGATGTGACGATCGTTGCGAATAGCACCGAA
GCGAGCCGCAAGAGCCTGTACGACCTGACCAAGAGCCTGGTGGCAACGTCCCAAGTTGAAGATCTGGTTGTT
AACCTGGTGCCGCTGGGTCGTTAA

dBroccoli genelet

GGAATGATAATACGACTCACTATAGGGAAGCCTGAGACGGTCGGGTCCATCTGAGACGGTCGGGTCCAGATATTCG
TATCTGTCGAGTAGAGTGTGGGCTCAGATGTCGAGTAGAGTGTGGGCTCAGGCTT

4 References

[1] S. R. MacEwan, W. Hassouneh, A. Chilkoti, *J. Vis. Exp.* **2014**, *88*, e51583.

[2] H. R. Marsden, L. Gabrielli, A. Kros, *Polym. Chem.* **2010**, *1*, 1512-1517.

[3] a) Y. Tamba, T. Tanaka, T. Yahagi, Y. Yamashita, M. Yamazaki, *Biochim. Biophys. Acta* **2004**, *1667*, 1-6; b) B. R. Casadei, C. C. Domingues, E. de Paula, K. A. Riske, *Biophys. J.* **2014**, *106*, 2417-2425.

[4] Z. Z. Sun, C. A. Hayes, J. Shin, F. Caschera, R. M. Murray, V. Noireaux, *J. Vis. Exp.* **2013**, e50762.

[5] M. Weitz, J. Kim, K. Kapsner, E. Winfree, E. Franco, F. C. Simmel, *Nat. Chem.* **2014**, *6*, 295-302.

[6] H. G. Döbereiner, J. Käs, D. Noppl, I. Sprenger, E. Sackmann, *Biophys. J.* **1993**, *65*, 1396-1403.
